# Mobile phones electromagnetic radiation and NAD+-dependent isocitrate dehydrogenase as a mitochondrial marker in asthenozoospermia

**DOI:** 10.1016/j.biopen.2016.09.001

**Published:** 2016-09-22

**Authors:** S.M.J. Mortazavi, S.A.R. Mortazavi, Maryam Paknahad

**Affiliations:** Medical Physics Department, School of Medicine, Shiraz University of Medical Sciences, Shiraz, Iran; Ionizing and Non-ionizing Radiation Protection Research Center (INIRPRC), Shiraz University of Medical Sciences, Shiraz, Iran; Student Research Committee, Shiraz University of Medical Sciences, Shiraz, Iran; Oral and Maxillofacial Radiology Department, School of Dentistry, Shiraz University of Medical Sciences, Shiraz, Iran

Dear Editor

With great interest, we have read the article by Hagras et al. entitled “Mobile phones Electromagnetic radiation and NAD+-dependent Isocitrate Dehydrogenase as a mitochondrial marker in Asthenozoospermia”, published in the journal Biochimie Open (2016), http://dx.doi.org/10.1016/j.biopen.2016.07.003 [1]. Hagras et al. investigated the possible relationship between mitochondrial NAD+-IDH activity in human seminal plasma and sperm motility among asthenozoospermic cellular phone users. The authors showed that IDH activity was increased in patients with prolonged cell phone daily use ≥4 h/day. Over the past several years, our team has conducted several studies on the possible association of mobile phone use and radiofrequency electromagnetic fields (RF-EMFs) health hazards [2], [3], [4], [5], [6], [7], [8], [9], [10], [11], [12], [13], [14]. Although the paper authored by Hagras et al. addresses a very challenging issue, it has some shortcomings. The first major shortcoming of this paper is due to lack of data about the basic parameters of exposure to RF-EMFs such as the specific absorption rate (SAR) of the mobile phones used by the participants or the average distance between the users' homes/workplaces and the nearest base stations (people who live in rural areas or remote locations with weak mobile phone signa1 strength will be exposed to higher levels of RF-EMFs because a much higher intensity of radiation is then emitted by the mobile phone to compensate the weak signal strength). SAR is the measure of the rate at which the energy of radiofrequency radiation is absorbed. SAR levels for mobile phones usually range from a minimum of about 0.20 to the maximum of 1.54 W/kg (The FCC limit for public exposure from mobile phones is 1.6 W/kg).

The second shortcoming of this study comes from this fact that the authors have not assessed whether the mobile phones were connected to the Wi-Fi network. It is worth noting that today mobile phones are much more frequently used for message exchange (texting) and surfing the Internet than for calling.

## Conflict of interests

The authors declared no conflict of interest.
